# Editorial: Biofilm and Food: Well- and Lesser-Known Interactions

**DOI:** 10.3389/fmicb.2022.923021

**Published:** 2022-05-09

**Authors:** Marilena Budroni, María-Jesús Torija, Jaime Moreno-García, Giacomo Zara

**Affiliations:** ^1^Department of Agricultural Sciences, University of Sassari, Sassari, Italy; ^2^Department of Biochemistry and Biotechnology, Oenology Faculty, Rovira i Virgili University, Tarragona, Spain; ^3^Department of Agricultural Chemistry, Edaphology, and Microbiology, University of Córdoba, Córdoba, Spain

**Keywords:** biofilm, spoilage microorganisms, protechnological microorganisms, wine, dairy industries

Microbial biofilms are highly structured microbial communities attached to a surface and enclosed within self-produced extracellular polymeric substances (EPS). Biofilm formation is considered a survival strategy that allows microbial cells to overcome many different stresses, among which high ethanol and salt concentrations, low pH, and low water activity. Thus, biofilms are tenacious structures that have different implications in food processing.

On the one hand, the biofilm formed by pathogenic and spoilage microorganisms on food products, or food contact surfaces, leads to serious hygienic problems and economic losses due to direct spoilage, cross-contamination, and post-processing contamination. In addition, biofilm cells are dramatically different from planktonic cells and display higher resistance to the commonly used sanitizers and antimicrobial agents. Considering biofilms as a reservoir of negative microbial species, recent research efforts have been directed toward the identification of proper sanitation strategies and novel inhibitory substances to eliminate negative biofilm in a more effective, economic, and sustainable way.

On the other hand, the formation of biofilms by pro-technological microorganisms is crucial for producing different foods. For example, the formation of biofilm by *Saccharomyces cerevisiae* is essential for the biological aging of some special wines, or acetic acid bacteria biofilm is sometimes required to produce vinegar. Thus, modern technological approaches are being developed to promote biofilm formation by positive microbial species to improve food quality.

In this context, this Research Topic aims to provide an overview of the recent studies on microbial biofilms, focusing on the identification of environmental effectors and innovative strategies to promote the dispersal and inhibition of health-threatening biofilms and on the study and characterization of positive biofilms. This Research Topic comprises 5 original articles (including 1 mini-review), contributed by 28 authors.

Three articles focused on the control of “negative” biofilms, such as those formed by pathogenic and spoilage microorganisms.

Kumar et al. summarized the current knowledge on the use of enzymes for the control and dispersal of bacterial biofilms in the dairy industry. Particularly, the authors aimed their attention at the mechanism of action of different enzymes against EPS components. Furthermore, the authors critically reported the research gaps, such as the proper identification of enzyme substrate specificity and thermal stability, which limit the adoption of enzymes as sustainable alternatives to chemical treatments during cleaning-in-place (CIP) treatments.

Similarly, Lin et al. dealt with the identification of reliable alternatives to physio-chemical treatments to prevent the formation of health-threatening bacterial biofilms. Particularly, the authors evaluated the use of the probiotic *Bacillus subtilis* natto against *Enterococcus faecalis*. The authors found that culture fluids of *B. subtilis* contain metabolic derivatives able to inhibit *E. faecalis* biofilm formation by reducing exopolysaccharide synthesis, restructuring its cell envelope, and downregulating the transcription of genes involved in peptidoglycan and membrane glycolipid biosynthesis. Results obtained suggest a direct mechanism of action of *B. subtilis* supernatant, in agreement with previous studies that reported the ability of *B. subtilis* to secrete molecules such as biosurfactants and bacteriocins, able to inhibit biofilm formation by pathogenic microorganisms.

Biofilm formation can also be reduced or inhibited *via* indirect mechanisms, such as changes in the physicochemical characteristics of the substrate. On this matter, Petrin et al. evaluated the effect of pH and salinity on the development of biofilm by 15 different *Salmonella* serotypes. This is of relevance in the food sector, as most food-borne infections are caused by only a few *Salmonella* serotypes. By developing a linear mixed effect model, the authors showed that while salinity was not effective, even at high concentrations, low pH values significantly influenced *Salmonella* biofilm-forming ability. These results suggest that only specific *Salmonella* serotypes may be associated with the colonization and persistence of biofilm cells in harsh conditions. Interestingly, the *Salmonella* serotypes causing the most human infections showed a limited ability to produce a biofilm.

As previously stated, microbial biofilms are required to produce specific foods, such as wine, cheese, olives, etc. From this perspective, two articles reported on the taxonomic composition and metabolite production of such beneficial biofilms in winemaking.

The paper by Carbonero-Pacheco et al. analyzed the yeast diversity in the biofilm developed on the surface of Sherry wines. The formation of a yeast biofilm, also known as *flor*, is essential for the aging and production of Sherry and Sherry-like wines, as yeast cells oxidize ethanol to acetaldehyde and produce metabolites peculiar to these wines. The main purposes of the study were to understand the complexity of the *flor* biofilm in terms of its taxonomic composition and to uncover the potential link between yeast species and the chemical profile of wines. By using novel culture-independent (ITS-metabarcoding) and culture-dependent (MALDI-TOF MS) techniques, the authors found that the biofilm on wine is composed of eight yeast species and that non-*Saccharomyces* yeasts have a low impact on the Sherry wine metabolome during aging. This last result is particularly interesting and contrasts with the well-known impact of non-*Saccharomyces* yeasts during the alcoholic fermentation of the grape must.

The significance of biofilm formation by *Oenococcus oeni* in the improvement of wine quality is the main aim of the paper by Tofalo et al. Even though *O. oeni is* the main bacteria involved in the malolactic fermentation of wine, very little is known about its ability to adhere to abiotic surfaces and the metabolic changes it undergoes during the biofilm phase. Tofalo et al. found that the ability of *O. oeni* to adhere to polystyrene plates is strain-dependent and that the cells detached from the biofilm increased their malic acid degradation kinetics and influenced the content of esters, higher alcohols, and organic acids in the final wine.

Hence, to conclude the whole Research Topic, the contributions received highlighted the dual role of microbial biofilms in the food industry and we are confident that the articles included in this Research Topic will inform the scientific community about the current knowledge, and challenges that still have to be overcome, about the appropriate measures to favor the formation of beneficial biofilms and to avoid the development of the negative ones.

## Author Contributions

All authors listed have made a substantial, direct, and intellectual contribution to the work and approved it for publication.

## Conflict of Interest

The authors declare that the research was conducted in the absence of any commercial or financial relationships that could be construed as a potential conflict of interest.

## Publisher's Note

All claims expressed in this article are solely those of the authors and do not necessarily represent those of their affiliated organizations, or those of the publisher, the editors and the reviewers. Any product that may be evaluated in this article, or claim that may be made by its manufacturer, is not guaranteed or endorsed by the publisher.

